# The flexion synergy, mother of all synergies and father of new models of gait

**DOI:** 10.3389/fncom.2013.00014

**Published:** 2013-03-13

**Authors:** Jacques Duysens, Friedl De Groote, Ilse Jonkers

**Affiliations:** ^1^Department of Kinesiology, KU LeuvenHeverlee, Belgium; ^2^Department of Research, Sint MaartenskliniekNijmegen, Netherlands; ^3^Department of Mechanical Engineering, KU LeuvenHeverlee, Belgium

**Keywords:** flexion reflex, local sign, reflex modules, synergy, central pattern generator, gait, forward model

## Abstract

Recently there has been a growing interest in the modular organization of leg movements, in particular those related to locomotion. One of the basic modules involves the flexion of the leg during swing and it was shown that this module is already present in neonates (Dominici et al., 2011). In this paper, we question how these finding build upon the original work by Sherrington, who proposed that the flexor reflex is the basic building block of flexion during swing phase. Similarly, the relation between the flexor reflex and the withdrawal reflex modules of Schouenborg and Weng (1994) will be discussed. It will be argued that there is large overlap between these notions on modules and the older concepts of reflexes. In addition, it will be shown that there is a great flexibility in the expression of some of these modules during gait, thereby allowing for a phase-dependent modulation of the appropriate responses. In particular, the end of the stance phase is a period when the flexor synergy is facilitated. It is proposed that this is linked to the activation of circuitry that is responsible for the generation of locomotor patterns (CPG, “central pattern generator”). More specifically, it is suggested that the responses in that period relate to the activation of a flexor burst generator. The latter structure forms the core of a new asymmetric model of the CPG. This activation is controlled by afferent input (facilitation by a broad range of afferents, suppression by load afferent input). Meanwhile, many of these physiologic features have found their way in the control of very flexible walking bipedal robots.

## Introduction

One of the first authors to point out the modular organization of the motor control system was Sherrington (1910a,b). Reading his work, it is clear that for him the flexor reflex was the mother of all modules and synergies (in the broad sense, not in the sense of the mathematical synergies defined recently). He proposed that the flexor reflex is a basic building block of the central nervous system and that “the flexion reflex is in reality the reflex stepping of the limb” (pp. 69 in Sherrington, 1910b). In his view, stepping was basically a series of flexion reflexes, with extension occurring merely as the “rebound” following the flexion. The extension during the stance phase of gait could be provided as some type of “extensor thrust,” evoked by “the weight of the animal applied through the foot against the ground” (pp. 78 in Sherrington, 1910b). In the absence of support (air stepping), the rhythmic activity continues, which for Sherrington was an argument for stating that “the extensor thrust cannot therefore be an indispensable factor in the reflex step” (pp. 79 in Sherrington, 1910b). This idea of a basic asymmetry in the control of locomotion has since lost terrain, mostly because of the powerful impact of the (symmetrical) half-center model for the central pattern generation of locomotion (one half of this center inducing activity in flexors, the other in extensors). The latter model was described by Brown (1914) and is known as the “half-center” model. The first ideas in that direction were actually presented by Sherrington himself on the basis of work by Brown (1911, 1912). They are based on experiments showing that cats with a transected spinal cord and with cut dorsal roots still showed rhythmic alternating contractions in ankle flexors and extensors. However, Sherrington did not necessarily propose a symmetrical organization. Instead, he and Brown proposed originally that gait was the result of a balance “between equal and opposite states of excitation” in flexors and extensors, while being well-aware that the origin of these states could be quite different. The latter notion seems to have been lost in later years.

In addition, in many cases the discussion on the credits for the original ideas about a central basis for locomotion has been simplified considerably in many accounts (as explained elegantly in a review by Stuart and Hultborn, 2008). Both Sherrington (1910a) and Philippson (1905) have indeed emphasized the idea that during gait one phase induced automatically the next one (reflex chain) but this does not mean that these authors excluded a central origin for the rhythmic activity (for details see also Clarac, 2008). In particular, Philippson believed that the spinal control was due to a combination of central and reflex mechanisms (Clarac, 2008). Hence it is a gross simplification to see this part of the history as a “victory” of Brown over his competitors (Sherrington and Philippson). Brown should be credited for having provided compelling evidence for the central spinal origin of locomotor activity, while Sherrington and Philippson should be remembered for their important insights on the importance of afferent input for the control of gait.

The “half-center” model has helped us greatly in appreciating the spinal origin of the central pattern generator (CPG) for locomotion, but it may have led to the simplifying idea of symmetry within the CPG (Jankowska et al., 1967a,b; Lundberg, 1981; Lafreniere-Roula and McCrea, 2005). In that way it has deterred our thinking away from the notion of a basic asymmetry of the neural organization of locomotion.

**Figure 1:**
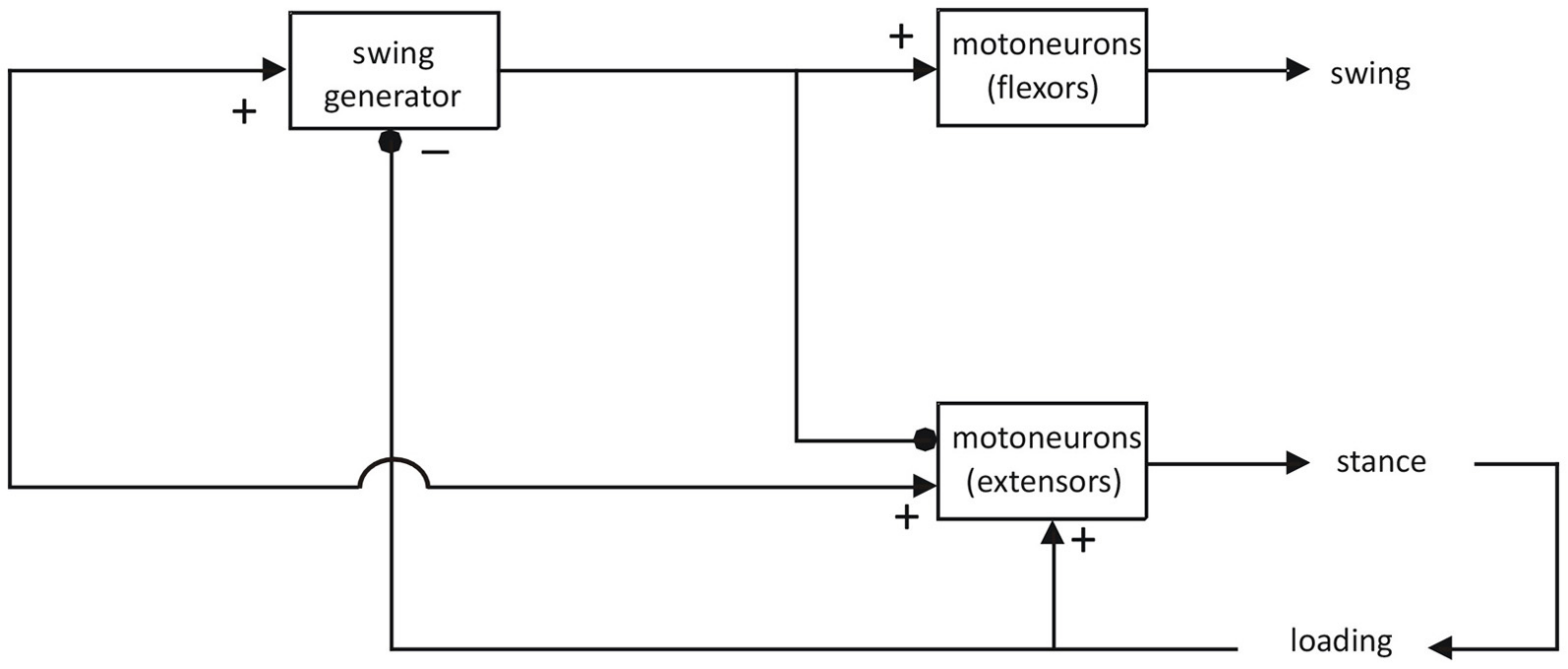
**Asymmetric model for the generation of locomotion.** Adapted from Pearson and Duysens (1976). This model could underlie a number of locomotor behaviors, as long as they include a flexor and an extensor phase. In humans, the question has been raised whether one should not assume that there are separate spinal CPGs for different types of gait, such as for forward and backward gait (Jansen et al., 2012) or for walking and running (Sylos Labini et al., 2011). In general, these studies are more in favor of the idea that the same CPGs can be utilized for different locomotor behaviors but that different supraspinal descending systems facilitate the reconfiguration of the spinal CPGs. This is in line with recent work on animal species where it is possible to record from individual neurons within CPGs (see “Discussion” in the papers mentioned above).

Nevertheless, there have been attempts to remediate these shortcomings and to explain gait in terms of asymmetric models (for review see Guertin, 2009). For example, Pearson and Duysens (1976) introduced a swing generator model, based on work on cats and cockroaches (Figure 1).

This model again assigns the flexor synergy (defined as the synchronous activation of flexors) a central place (“swing generator”). In contrast, the activation of extensors is thought to rely more on feedback systems, notably for load receptors (Duysens and Pearson, 1980; Dietz and Duysens, 2000; Duysens et al., 2000; Pearson, 2004). For the flexor synergy, there is little doubt that there may be an involvement of part of the spinal CPG for locomotion, even for humans (Duysens and Van de Crommert, 1998). However, for the extensor synergy, the requirements are very different. The extensor synergy is called upon by the loading of the limb. Pressure on the foot sole can simulate this loading and results in an “extensor thrust” (Sherrington, 1906). Hence, it is basically a peripherally driven synergy, not a centrally triggered one. In terms of sensory feedback the organization of gait is basically asymmetrical since interaction with the environment is much more intense during the stance phase (Duysens, 2006). In contrast, for the swing phase, there is only the need for a trigger (in this case limb unloading and hip extension). Even Sherrington already recognized that the flexor synergy was greatly facilitated by hip extension (see pp. 81 in Sherrington, 1910b). For him, the extension phase followed automatically after the flexion phase, which was the only phase that needed to be centrally triggered. From earlier cat work, it is confirmed that this transition to the stance phase is indeed facilitated at the end of the flexor activity (Duysens, 1977).

Recent data have provided support for such asymmetric models. Thanks to the insights from recent use of genetic manipulations of CPG neurons, it is now widely accepted that the core premotor components of locomotor circuitry are common and derive from a set of embryonic interneurons that are remarkably conserved across different species (e.g., Goulding, 2009). In particular, it is of interest to consider the organization of “swimming” CPGs since they are the evolutionary basis for the “walking” CPGs. In this respect, it should be emphasized that these models of the swimming CPG are highly asymmetric. In the lamprey, for example, there are four functional classes of neurons in the swimming CPG. One of these four consists of excitatory glutamatergic neurons (EINs), projecting to all three other CPG neuron cell types. These cells provide rhythmic drive to other CPG neurons during swimming.

In mammalian systems the idea of an asymmetric CPG is also taken seriously (Brownstone and Wilson, 2008; Zhong et al., 2012). Some of the evidence relies on the observation that rhythmic bursts of activity (in muscles or nerves to leg muscles) sometimes are skipped during periods of real or fictive locomotion (this is termed “spontaneous deletions”). They often occur in reduced preparations of cats (Duysens, 1977, 2006) or rats (Zhong et al., 2012). Such deletions are hard to explain on the basis of a simple half-center model (McCrea and Rybak, 2007, 2008). One typical feature is that these deletions are highly asymmetric: flexor deletions are accompanied by sustained ipsilateral extensor activity, whereas rhythmic flexor bursting is not altered during extensor deletions. Such results are best explained by a rhythm generator that provides direct input to a “swing” or “flexor burst” generator but not to the extensor part of the CPG (Rybak et al., 2006a,b; Zhong et al., 2012). Hence, it is basically similar to the model proposed originally by Pearson and Duysens (1976) except that the swing generator is split up in a rhythm generator and a flexor center.

Some earlier cat modeling work had pointed toward asymmetry as well. For example, the model proposed by Prochazka and Yakovenko (2007a) seems symmetrical at first sight but it already contains important elements of asymmetry. In particular it is argued that interneurons in the extensor timing element may receive less inputs generating persistent inward currents, therefore as a network “they are not only set to have longer half-cycle durations, but also to be more sensitive to synaptic commands.” Interestingly, the model was only stable for extensor dominant phase-duration characteristics (where extension durations vary more than flexion durations; see also Prochazka and Yakovenko, 2007a,b). This pattern is seen in the normal cat. The inverse (flexor dominant) was, however, not observed in the model while it has been observed experimentally, but only in fictive locomotion (rhythmic output of the spinal cord in paralyzed cat preparations). This is an important point as the existence of both flexor- and extensor-dominated patterns has often been invoked to support the notion of a symmetrical CPG (McCrea and Rybak, 2008). However, one may wonder whether the flexor-dominated pattern is not simply an artifact of the preparation used, since the output observed is one from CPGs without interaction with afferent input. As pointed out above, the afferent input is crucial for the automated phase transitions. Experimental work on cats has clearly established that peripheral input from the paw (as occurs during touchdown) is very potent in terminating the flexor phase and initiating the extensor phase (Duysens, 1977). Hence, in the absence of such feedback it is not surprising to see flexor phases of abnormally long duration.

This feature was not always recognized and perhaps for this reason, the concept of an asymmetric model first met some resistance (McCrea and Rybak, 2007, 2008). However, due to more recent data (Brownstone and Wilson, 2008; Zhong et al., 2012), the idea of an asymmetric pattern generator has reemerged and it is therefore worthwhile to reexamine the presumed basis of the swing generator, namely the flexor synergy, its adaptations (for example to stimulation of different skin areas on the leg, a phenomenon referred to as “local sign” in physiology) and its integration in the process of locomotion.

## The task to withdraw and the corresponding flexor synergies in the spinal cord: a defense in favor of the “local sign”

For the flexor reflex, it is clear that the synergy (as described by Sherrington) corresponds very well to the task of withdrawal. This protective reflex is so important that it is present at birth and can be elicited with about any type of stimulus to the foot. In neonates, the flexion reflex responses to innocuous stimulation are already present (Andrews and Fitzgerald, 1999). How do these responses compare to the more recently described synergies (or “components”)? In the adult, the flexor reflex cannot simply be related to just one component, although factor 5, as described by Ivanenko et al. (2004), or P3 as described by Dominici et al. (2011), are close candidates. For example, the factor 5 of Ivanenko et al. (2004) relies of strong activations of Sartorius and Tibialis Anterior during the middle of the swing phase.

In neonates, the gait is explained (up to 89%) by just two patterns, one of which peaks at about 75% of the step cycle, hence in the swing phase. This “swing” pattern persists in the adults and is seen in a wide variety of species. When one considers the large input of flexors to these basic patterns, it is tempting to relate these components to the flexor synergy as described by Sherrington (1910b). Furthermore, the appearance of these components in swing is nicely in line with the Sherrington proposal of a common neural basis for the flexor reflex and the flexion phase of stepping. Further experimental evidence for such common use of neural circuitry has been obtained in animal studies. For example, in the turtle, Berkowitz has described interneurons that are active in both types of activity (flexion phase and flexor reflex; Berkowitz, 2007, 2010). In addition, in the same species it was shown that often the same interneurons can be involved in various types of rhythmic behavior (swimming, scratching), thereby supporting the idea that basic synergies can be used in various behaviors (Berkowitz and Hao, 2011; see also Grillner, 1985).

During maturation in humans, the threshold for the reflex increases and biceps femoris responses dominate (Andrews and Fitzgerald, 1999). Furthermore, the pattern of the flexor reflexes changes. The recruitment of specific flexor muscles depends increasingly more on the area of skin stimulated (“local sign”), thereby allowing a more efficient withdrawal when stimuli are applied at various distinct locations on the limb. This has led several authors to propose the existence of a variety of reflex modules both in humans (Andersen et al., 1999, 2001; Sonnenborg et al., 2000, 2001) and in animals (Schouenborg and Weng, 1994; Tresch et al., 1999).

There is no doubt that these new experiments have provided a wealth of very precise data but still the question arises whether this has basically altered our way of thinking. The idea of a “local sign” goes back to the early days of reflex physiology. Creed and Sherrington (1926) stated (pp. 265): “The term flexion-reflex … denotes strictly speaking a group of reflexes, all more or less alike … yet from one afferent to another differing in detailed distribution of the motor units employed, while yet always conforming to the general type flexion-reflex.” Especially this last point is important as it is proposed originally that there remains a basic synergy (“flexion-reflex”) underlying all these different variations. The data of Creed and Sherrington (1926) showed that, despite variations in some of the distal flexors, the hip and knee flexors always participated in the various reflexes (see their table on pp. 260). More recent work supports this, both in the frog (Tresch et al., 1999) and in the rat (Schouenborg and Kalliomaki, 1990). However, this common element is often not emphasized and the impression may arise that the recently defined “modules” and “synergies” are independent entities. This certainly differs from the view of Creed and Sherrington (1926), who viewed the different versions of the flexor reflexes as expression or adaptations of one and the same basic flexor synergy. Hence the basic issue is whether the recently described reflex modules are also mostly variations of a basic synergy (the flexion reflex) or whether they really constitute separate entities.

In our opinion, there is no convincing evidence for the latter, at least when one considers the literature on withdrawal reflexes. Local cutaneous reflexes do exist, but they differ from withdrawal reflexes. For example, the selective activation of extensor reflexes such as the gastrocnemii was observed when stimulating the skin that covered these muscles (Hagbarth, 1960). For withdrawal reflexes, in contrast, there is no data to show convincing evidence for neural pathways for separate types of flexor reflexes. They mainly show modifications of a basic flexor pattern. These modifications are likely to be due to changes in activity in spinal dorsal horn cells (Schouenborg et al., 1995). During development, the withdrawal reflexes are “fine-tuned” by the spontaneous movements of the individual but the resulting reflexes always have a component of hip and/or knee flexion (Holmberg and Schouenborg, 1996). Hence these studies provide a substantial contribution to our knowledge on the “local sign” but they do not show that there is a conceptual deviation from the notion of “local sign,” as originally defined. In addition these studies underline the plasticity of reflexes. Synergies, as defined more recently in mathematical terms, do not fully overlap with these reflexes. Nevertheless, several authors have emphasized that these muscle synergies and modules may also be highly plastic and basically represent solutions for specific tasks at a given time (Latash, 1999; Ivanenko et al., 2013).

## Pathology

The data provided by pathology further support the notion of variations in flexor reflexes rather than a set of separate modules. As one might expect, the adjustments and fine-tuning of the flexor reflex relates to input from descending pathways. Hence, when a lesion occurs in these pathways, one should see a reversal to the more primitive state. This is exactly what happens.

In spinal cord injury (SCI) there is a loss of “local sign” and a return to the simpler forms of flexor reflexes (Schmit et al., 2003; “an invariant flexion response pattern was produced regardless of stimulus location”). In addition, in these patients there is a link between a normal flexor reflex and the ability to recover gait (Dietz et al., 2009). After some 6–12 months, this ability deteriorates when the early flexor reflex (latency 60–120 ms) decreases over time. Again, this illustrates the importance of the flexor reflex circuitry for the generation of gait. In stroke, a similar return to a more primitive synergy occurs after the insult and this phenomenon is known as the Babinski sign (Babinski, 1922). Stimulation of the sole of the foot induces dorsiflexion of the big toe, by activating the extensor hallucis longus muscle, a flexor in the physiological sense. Babinski pointed out that this reflex was part of the flexion synergy of the lower limb and in fact clinicians, still as of today, are advised to watch for flexion of the whole limb as an obligatory concomitant of the reflex (Van Gijn, 1978; Kumar, 2003). The whole reflex is a return to the condition of the neonate, where indeed a dorsiflexion Babinski is normally present, usually in conjunction with a brisk flexion of the whole limb. Interestingly, in the neonate it is important not to stimulate too gently, because otherwise a grasp reflex occurs. This shows that actually what we know as the “normal plantar reaction” (plantar flexion of the toes) may actually be a superposition of two reflexes, with the grasp reflex dominating the flexor reflex. This makes sense in an evolutionary context since, for example, grasping tree branches might have been more important than a “blind” withdrawal defense toward any type of stimulus. In complete SCI subjects, the occurrence of the Babinski sign has been described as well, although it can be absent in some patients due to associated peripheral nerve damage (Petersen et al., 2010).

## Can the real flexor reflex please stand up!

One problem in this research field is the confusion on the flexor reflex terminology. In humans, most studies do not use pure nociceptive stimuli such as heat. Instead, electrical stimulation is used. However, it is impossible to activate nociceptive afferents in any nerve without coactivating large myelinated fibers. Therefore, in humans, the response to high intensity electrical stimuli typically has two components, an early (60–120 ms) and a late one (120–200 ms; Shahani and Young, 1971). The difficulty is to decide which afferents are responsible for a given component. If only high intensity stimuli are used, one is easily misled in thinking that the early response is a nociceptive one, while in fact it often can be elicited by low intensity stimuli as well. The problem is aggravated by differences in definition. Hugon (1973) defined the early response (RII) as having a latency of 40–60 ms and the late response (RIII) as having a latency of 85–120 ms (for review see Sandrini et al., 2005). Hence, RIII is really the equivalent of the “early” flexor reflex. In normal control subjects, walking on a treadmill, one can easily evoke RIII responses in a variety of muscles with stimuli that are just above perception threshold (Duysens et al., 1990). Nevertheless, some people label this component as “the flexor reflex” and in fact it was even claimed to be useful as an index of pain (Willer, 1977).

Part of the problem is that some of these reflexes are also task-dependent, needing stronger stimulation under unfavorable conditions. For example, the RIII component can be elicited very easily by stimulation of non-nociceptive low threshold afferents during gait while the same responses may be small or absent in subjects at rest (Duysens et al., 1993; Komiyama et al., 2000). During gait, the RIII responses are especially prominent in muscles such as biceps femoris and tibialis anterior both in intact cats (Duysens and Loeb, 1980) and in intact humans (Duysens et al., 1990; Yang and Stein, 1990; Zehr et al., 1997). When cutaneous stimuli are given at the ankle just prior to the onset of the swing phase, they elicit responses in these flexors, just as one would expect from Sherringtons' work (see Duysens et al., 2004). However, at end of swing the same stimuli elicit facilitatory responses in extensor muscles (Duysens et al., 1990) while providing suppression to flexor muscles (Duysens et al., 1990; Yang and Stein, 1990). This has been termed “reflex reversal” (in analogy with the use of this term in cat literature, Forssberg et al., 1975; Duysens and Pearson, 1976). In later work it was shown that such reversal of EMG responses resulted in a reversal of behavioral responses (flexion, extension) as well (Duysens et al., 1992; Zehr et al., 1997). Furthermore, the responses depended heavily on “local sign” (Van Wezel et al., 1997; Zehr et al., 1997, 1998; Nakajima et al., 2006).

These examples show that synergies are extremely flexible and their expression depends highly on the task and the phase of the movement (“phase-dependent modulation”). A given stimulus does not always elicit the same responses in the same muscles. One way to interpret this type of results is by assuming that a given afferent input (or descending command) is translated in the spinal cord into responses that are appropriate for the state of the interneurons related to a given phase of the movement (Drew, 1991). This view differs from the contention that reflexes or synergies are fixed building blocks. Instead it opens the way to the idea that they are highly adaptable entities depending on the constraints of the environment and the state of the central nervous system (“time-varying muscle synergies,” d'Avella et al., 2003; Ivanenko et al., 2006b).

An important unresolved issue concerns the pathways of the flexor reflexes or synergies. Since both components of the flexor reflex persist in patients with a complete spinal cord lesion, it is evident that the minimal responsible pathways could go through the spinal cord (Shahani and Young, 1971). In fact, in recent literature the first component is often simply labeled “spinal reflex” (Dietz et al., 2009; Bolliger et al., 2010; Dietz, 2010; Hubli et al., 2011, 2012). While this is entirely appropriate for SCI patients, it can be questioned whether this can also be used as a valid term when intact humans are tested since responses with similar latencies have been related to circuits either through brainstem (spinobulbospinal “SBS” reflexes, Shimamura et al., 1980) as well as through cortex (Christensen et al., 1999). Hence, the responses at a given latency can arise from very different sources.

## Acting against gravity: extensor synergies in the spinal cord

In the interaction with the environment, one of the most crucial forces to deal with is gravity. This even applies to the flexor reflex. Indeed, it is often overlooked that the flexor reflex involves not only the activation of flexor muscles but also the suppression of extensor activity. This could be particularly important for situations where the limb is loaded, for example during the stance phase of gait. In such cases it is crucial that a contact with a nociceptive stimulus (a sharp object) can induce a fast unloading of the limb (Santos and Liu, 2007). However, in most cases with normal ground surface, there is no need for unloading but instead there is a need to recruit additional extensor activity as soon as the limb is loaded (early stance). In the latter case, there is a need to suppress the flexor synergy. Work on cats has revealed that this is achieved through the activation of load receptors in the extensor muscles (Duysens and Pearson, 1980; Whelan, 1996; Duysens et al., 2000). Models, allowing reinforcing feedback from extensors during the stance phase of gait, have successfully simulated cat gait (Prochazka et al., 1997). In humans, the role of load feedback in shaping the extensor output during gait has been recognized as well (Dietz and Duysens, 2000). Under conditions of simulated reduced gravity, even minimal contact forces, and a very limited amount of loading during the stance phase, have profound effects since it completely restores normal limb trajectory (Ivanenko et al., 2002).

In recent work, the activation of various extensors in the stance phase is identified as a synergy, based on a mathematical decomposition of the EMG data (factors 1 and 2 in Ivanenko et al., 2004; see also Ivanenko et al., 2006a,b, 2007, 2008). Consistent with the idea of combinations of synergies to simplify motor control, the combination of these patterns with other synergies leads to the full process of walking, (d'Avella et al., 2003; Lacquaniti et al., 2012). During maturation there is a gradual transition from a two synergy state control of gait (flexor extensor, in neonates) to a four state synergies in toddlers (Dominici et al., 2011) This is consistent with the idea that additional tasks (such as equilibrium control) are achieved by the addition of extra synergies. In this context, it is of interest that the synergy approach has also been applied successfully in studies on balance and posture (Ting and Macpherson, 2005; Torres-Oviedo et al., 2006). For gait, these synergies are well-established (Ivanenko et al., 2005) and they have been shown to be robust in a wide variety of gait conditions (Ivanenko et al., 2004, 2006a,b, 2007). In fact, it is now possible to use these synergies to model human gait (see below) and in the future it is conceivable that these new notions enter the field of robotics, since there is increasing interest to incorporate physiological features in the design of walking robots (Klein and Lewis, 2012).

## Introducing synergies in models of human gait

The question arises whether synergies can help to achieve a closer correspondence between calculated and experimentally measured muscle activity in models of human gait. Although it is recognized that muscle activity patterns underlying gait originate from a highly flexible modular system, this is largely ignored in simulation frameworks aiming to causally relate muscle action to gait kinematics and kinetics. Due to the redundancy of the musculoskeletal system, a single motion can be obtained by different muscle coordination strategies. Typically, a performance criterion is optimized to predict the muscle coordination strategy underlying a given motion. Static optimization algorithms minimize muscle activity while imposing that the corresponding muscle forces produce the net joint torques calculated using inverse dynamics (Anderson and Pandy, 2001). Although such optimization approaches predict some basic features seen in the muscles' EMG, other features are not well-predicted. Hence, in addition to biomechanical constraints, it is important to take the principles of neural control into account when estimating muscle activations (Ting et al., 2012). Recently, simulated gait motions based on modular activation patterns were successfully produced (Neptune et al., 2009; Allen and Neptune, 2012; Sartori et al., 2012). Neptune et al. (2009) use five muscle activation modules identified from EMG and assigned each muscle to one module. They then used an optimization approach to find the magnitude and timing of the activation patterns that minimized the tracking error in a forward simulation of gait. They found that the five modules framework they proposed can successfully simulate 2D walking but that it does not provide all control needed for 3D walking. This additional control is important since it may underlie the transition from neonate to adult walking (Dominici et al., 2011; see above).

**Figure 2:**
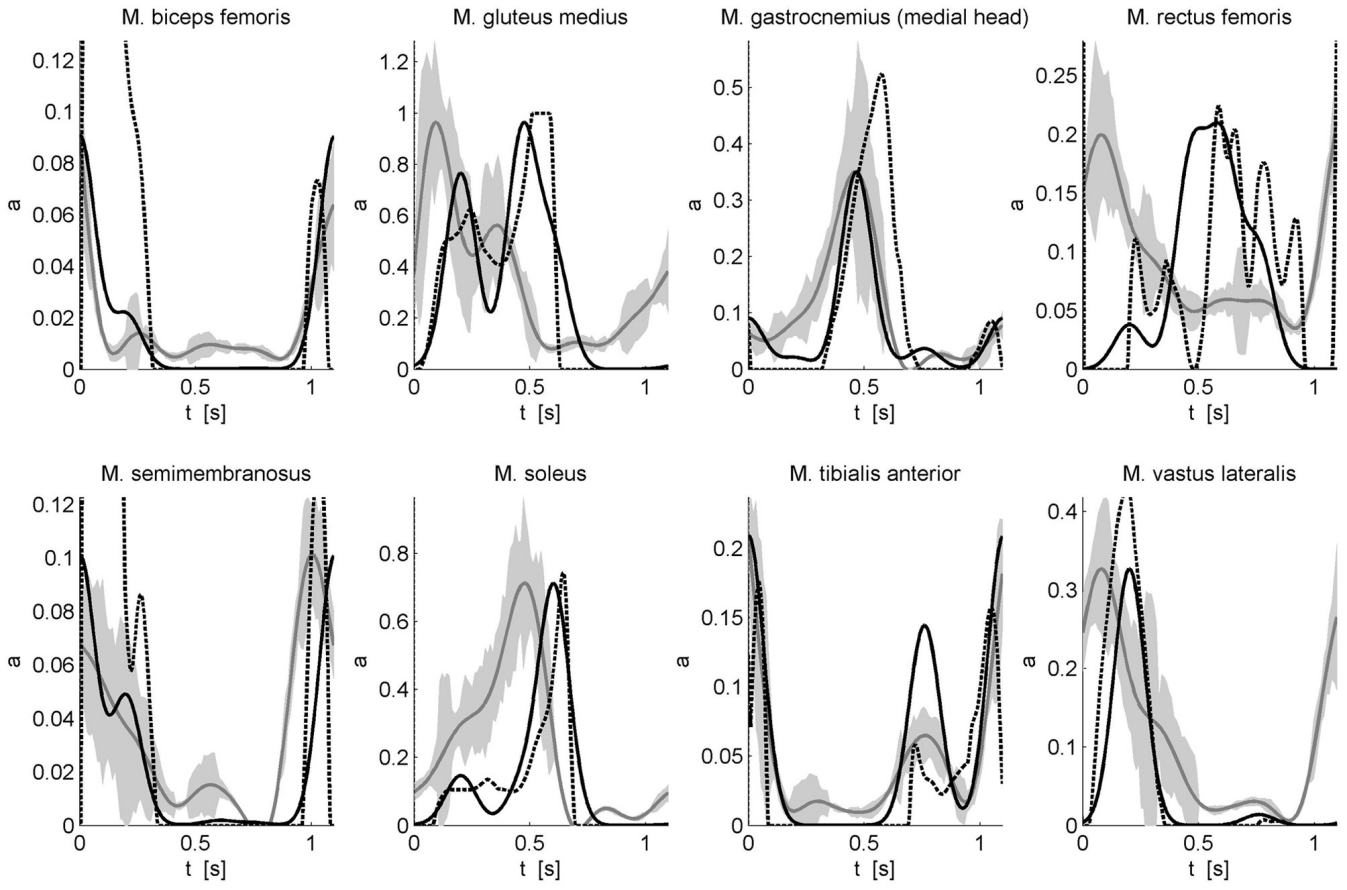
**Comparison of calculated activations and measured EMG for eight superficial muscles.** Activations underlying an experimentally measured gait motion were calculated using static optimization without any constraints on the activation pattern (dashed black) and by modeling the activation patterns as a weighted sum of Gaussian modules (solid black). EMG was measured for eight superficial muscles using surface electrodes. The EMG (solid gray with standard deviation indicated by the gray band) is scaled to the maximal modules-based activation. For more details on the experimental protocol and data processing see De Groote et al. (2012).

Alternatively, and in contrast to Neptune et al. (2009), an inverse approach can be used that allows each module to contribute to the activation pattern of all muscles, as described below. Ivanenko et al. (2006a) showed that during gait five Gaussian components G_*k*_(*t*) with a standard deviation of 6% of the gait cycle duration and appropriate timing account for 90% of the EMG variation. This representation was used to model muscle activation patterns underlying locomotion with each individual muscle activation pattern a_*m*_(*t*) described as a weighted sum of Gaussian components:

$${\text{a}}_{m}\left(t\right)=\sum {\text{w}}_{mk}\;{\text{G}}_{k}(t),$$

with w_*mk*_ the weight of muscle *m* for component *k*. This description of muscle activation patterns with a static optimization approach allows calculating muscle activations underlying a previously measured gait motion. However, in this approach, the timing of the Gaussian components and the muscle-specific weights of these components were determined using an optimization procedure minimizing the sum of muscle activations squared while a penalty term was used to impose that the corresponding muscle forces produce the net joint torques. The resulting activation patterns were compared to the solution of a “classic” static optimization approach without any constraints on the activation pattern and the measured EMG patterns (Figure 2). The experimental protocol, data processing including inverse dynamics, and the static optimization approach are described in De Groote et al. (2012).

The optimized timing of the Gaussian components is 18, 42, 55, 69, and 100% of the gait cycle. The differences between the calculated timings and the timings proposed by Ivanenko et al. are 8, 3, 0, 6, and 5%, respectively. The key features of the EMG are well-predicted by the modules-based activations. Although the correspondence with the inverse dynamics joint torques is higher when the activation patterns are not constraint to a weighted sum of Gaussians, the modules-based activations better predict the measured EMG of biceps femoris, gastrocnemius, and tibialis anterior. For other muscles such as soleus, gluteus medius, semimembranosus, and vastus lateralis there are still differences in timing. Based on preliminary results we feel that this can be improved by adding positive force feedback to the simulation. Finally for M. rectus femoris (RF) the fit is poor. The weak correspondence between measured EMG and calculated activations for RF is seen in both activation patterns and may be related to the notorious problem of cross-talk for surface EMG for this muscle (Nene et al., 1999, 2004). In fact, it has been recognized that cross-talk can affect synergies as well, but only to the degree that weighting coefficient are altered (Ivanenko et al., 2004). Therefore, some authors have insisted on using fine wire EMG recordings (Ivanenko et al., 2004). Another reason for the difficulty of modeling RF is that this muscle presumably has activity which depends heavily on afferent input and reflexes. For example, in cats the activity in RF differed between fictive locomotion (i.e., in absence of reflexes) and normal forward level walking, indicating that afferent input helps shaping the activity profile of this muscle during locomotor activity (Markin et al., 2012).

## Conclusions

It is clear that the synergy approach is very fruitful and that it can improve our understanding of human gait and its models, including new asymmetrical models of the CPG. Furthermore, it can be helpful in providing the basis of new neuro-computational approaches, as was shown here, as a proof of principle, for inverse dynamic calculation of muscle activations. Nevertheless, as concerns the popular notion of independent modules, a word of caution is in place since it is not fully appropriate to depict the modular organization as being a replacement of some of the older theories (“local sign”), such as those put forward by early physiologists (Creed and Sherrington, 1926).

### Conflict of interest statement

The authors declare that the research was conducted in the absence of any commercial or financial relationships that could be construed as a potential conflict of interest.
